# Opportunities and obstacles in harnessing intraoperative data

**DOI:** 10.3389/jaws.2026.16301

**Published:** 2026-06-02

**Authors:** Amanda Hernandez, Jayson Marwaha

**Affiliations:** 1 University of Michigan Medical School, Ann Arbor, MI, United States; 2 Department of Surgery, University of Michigan, Ann Arbor, MI, United States

**Keywords:** artificial intelligence, artificial intelligence (AI), intraoperative data, robot assisted surgery, robotic surgery

## Introduction

Robotic surgery utilization has increased rapidly over the past decade, a trend that is well recognized across surgical specialties, with general surgery representing the largest and fastest-growing contributor [1]. This expansion has been reflected by the transition of several complex general surgical procedures—such as pancreatectomy, colorectal surgery, and esophagectomy—from laparoscopic to predominantly robotic approaches [2]. Similarly, minimally invasive hernia repair has seen substantial growth, with robotic inguinal and ventral hernia repairs increasing more than 40-fold between 2012 and 2018 [2, 3].

A consequence of the rapid expansion of robotic surgery is the generation and capture of novel, high-dimensional data. The introduction of robotic platforms in the OR offers the potential to generate and capture rich intraoperative data streams, including modalities rarely or never captured previously, such as video, haptics, kinematics, audio, robotic usage metadata, and more. With the advent of this data, the previous “black box” of the intraoperative period has the potential to become more measurable and analyzable. High-fidelity and time-stamped data with synchronized audiovisual information collected over time can create large datasets that can ultimately be linked to clinical outcomes and identify best practices.

## Opportunities provided by robotic surgery data

The availability of this data provides new opportunities for improvements in patient care, education, quality improvement, innovations in intraoperative AI, and more. Current research on intraoperative AI applications is in early developmental stages, demonstrating how AI applications can enhance surgical precision through motion analysis as well as provide decision support through surgical phase recognition and anatomical landmark identification [4, 5], but this intraoperative data will surely prove to be instrumental in advancing the capabilities of these tools. Most existing intraoperative AI systems have the capability to provide simple assistance, with very few (3%) providing conditional decision-making [5, 6]. However, as higher-quality, task-specific intraoperative data are collected and analyzed, these data may help catalyze advancement of efforts to enable robots to perform select autonomous surgical tasks [7].

### Quality improvement

Data generated and collected on robotic platforms may also aid in quality improvement efforts in the form of retrospective analyses to ensure best practices are being met. For example, intraoperative data has already been used to ensure the critical view of safety is being achieved during a cholecystectomy or using ambient operating room sensors to collect audiovisual data to ensure a surgical timeout is always being performed [8, 9]. Metadata generated by how physicians interact with surgical technology contains valuable signals for understanding and improving healthcare delivery. For example, studying audit log data from how physicians use electronic health records has provided great insight into understanding how patient care is delivered [10, 11]. Thus, studying metadata from how users are interacting with the robot could give tremendous insight into how to improve intraoperative processes.

### Clinical research and education

On the side of clinical research, the data generation of robotic surgery has created a rapid pace of evidence development. This has encouraged increased research in the form of retrospective studies and database reviews [3]. The abundance of automated data generation unique to robots creates large and easily accessible datasets that allow researchers to analyze observational data. However, there is a need for well-designed prospective and randomized studies to create higher-quality evidence of the impact of this data.

### Education

In the context of education, data leveraged from robotic surgery can play a role in structuring surgical development, assessing skills, and monitoring performance improvement. Automated metrics derived from robotic tools can provide objective data on technical parameters, motion, and time to help trainees improve [12]. Data-driven insights from robotic surgeries can offer opportunities for standardized, objective skills assessment and feedback, supporting surgical education [13, 14].

## Opportunities and obstacles

These are among some of the many opportunities robotic surgery has to offer. There remain obstacles that prevent its true potential from being unlocked. The potential of this data and the involvement of multiple stakeholders in generating this data raise the issue of how this data should be governed so that it can be used for all of the potential benefits listed above. While not comprehensive, some important elements of establishing a governance infrastructure for this data are standardization and interoperability. Presently, there is a lack of a standardized and universally adopted ontology for intraoperative data in robotic surgery. Several groups, including the Society of American Gastrointestinal and Endoscopic Surgeons (SAGES, as well as industry groups such as Intuitive Surgical, are working to advance the ontology and annotation of intraoperative events through the development of standardized frameworks for surgical phases, tasks, actions, and gestures [15]. Standardized frameworks that exist outside of surgery can serve as models. A standard used in radiology called Digital Imaging and Communications in Medicine (DICOM) aims to ensure interoperability between different manufacturers’ devices and set guidelines for managing, storing, and transmitting medical image data [16]. Data standards adapted to intraoperative data could similarly be beneficial.

Additionally, data interoperability - the seamless transfer and aggregation of data across vendors, researchers, and healthcare systems - will be an important feature of this data to ensure it can be used productively. Interoperability is complementary to standardization: while standardization ensures data is in a comparable format across various vendors and institutions, interoperability ensures that there are resources to allow the data to be aggregated into larger datasets and used for more powerful analyses. As an example, the development of interoperability in the setting of EHR data may offer lessons for what this might look like for intraoperative data. There is a well-developed regulatory environment around the sharing of electronic health information (EHI), which is data commonly found in EHRs.The 21st Century Cures Act established rules around “information blocking” that prevent healthcare providers, health information technology developers, exchanges, and networks from interfering with authorized access to EHI [17]. The Office of the National Coordinator for Health Information Technology (ONC) Final Rule implemented these provisions and specified that there must be standardized application programming interfaces (APIs) that enable data sharing [18]. Finally, interoperability standards, including Health Level Seven (HL7) and Fast Healthcare Interoperability Resource (FHIR) were established to facilitate the integration, exchange, sharing, and retrieval of EHI [19].

## Conclusion

The rapid expansion of robotic surgery means that there has been an influx of new digital technology into the operating room that is capturing and generating surgical data that has never been done at this scale. There is great potential in using this data as it can be crucial to surgical skill improvement, patient care, and research efforts to improve surgical outcomes. A rate-limiting step to harnessing this data is to proactively ensure it is standardized and shareable. This is the first step to ensure a collaborative and cohesive environment that promotes innovation and inspires researchers and surgeons to optimize surgical care.

## References

[1] Sheetz KH Claflin J Dimick JB . Trends in the adoption of robotic surgery for common surgical procedures. JAMA Netw Open (2020) 3:e1918911. 10.1001/jamanetworkopen.2019.18911 31922557 PMC6991252

[2] Bonner SN Thumma JR Dimick JB Sheetz KH . Trends in use of robotic surgery for privately insured patients and medicare fee-for-service beneficiaries. JAMA Netw Open (2023) 6:e2315052. 10.1001/jamanetworkopen.2023.15052 37223903 PMC10209745

[3] Muaddi H Hafid ME Choi WJ Lillie E de Mestral C Nathens A Clinical outcomes of robotic surgery compared to conventional surgical approaches (laparoscopic or open): a systematic overview of reviews. Ann Surg (2021) 273:467–73. 10.1097/SLA.0000000000003915 32398482

[4] Cizmic A Mitra AT Preukschas AA Kemper M Melling NT Mann O Artificial intelligence for intraoperative video analysis in robotic-assisted esophagectomy. Surg Endosc (2025) 39:2774–83. 10.1007/s00464-025-11685-6 40164839 PMC12041040

[5] Vasey B Lippert KAN Khan DZ Ibrahim M Koh CH Layard Horsfall H Intraoperative applications of artificial intelligence in robotic surgery: a scoping review of current development stages and levels of autonomy. Ann Surg (2023) 278:896–903. 10.1097/SLA.0000000000005700 36177855 PMC10631501

[6] Lee A Baker TS Bederson JB Rapoport BI . Levels of autonomy in FDA-cleared surgical robots: a systematic review. NPJ Digit Med (2024) 7:103. 10.1038/s41746-024-01102-y 38671232 PMC11053143

[7] Kim JW Chen JT Hansen P Shi LX Goldenberg A Schmidgall S SRT-H: a hierarchical framework for autonomous surgery *via* language-conditioned imitation learning. Sci Robotics (2025) 10:eadt5254. 10.1126/scirobotics.adt5254 40632876

[8] Campbell KK Abreu AA Zeh HJ Daniel WC Palter VN Bishop SJ Using OR black box technology to determine quality improvement outcomes for *in-situ* timeout and debrief simulation. Ann Surgery (2026) 283:122–9. 10.1097/SLA.0000000000006438 38989569

[9] AI for automated detection of the establishment of critical view of safety in laparoscopic cholecystectomy videos. J Am Coll Surgeons 231, e48 (2020).

[10] Adler-Milstein J Adelman JS Tai-Seale M Patel VL Dymek C . EHR audit logs: a new goldmine for health services research? J Biomedical Informatics (2020) 101:103343. 10.1016/j.jbi.2019.103343 31821887

[11] Biases in electronic health record data due to processes within the healthcare system: retrospective observational study. BMJ 363, k4416 (2018). 30337282 10.1136/bmj.k4416

[12] Shehata D Ghaderi I Nepomnayshy D . The evolution of surgical skills simulation education: advanced laparoscopic skills. Surgery (2025) 181:109248. 10.1016/j.surg.2025.109248 39952019

[13] Jogerst KM Coe TM Petrusa E Neil J Davila V Pearson D Multidisciplinary perceptions on robotic surgical training: the robot is a stimulus for surgical education change. Surg Endosc (2023) 37:2688–97. 10.1007/s00464-022-09708-7 36414871

[14] Zhao B Lam J Hollandsworth HM Lee AM Lopez NE Abbadessa B General surgery training in the era of robotic surgery: a qualitative analysis of perceptions from resident and attending surgeons. Surg Endosc (2020) 34:1712–21. 10.1007/s00464-019-06954-0 31286248 PMC6946889

[15] Mlambo B Shields M Bach S Bauer A Hung A Kudsi OY A standardized temporal segmentation framework and annotation resource library in robotic surgery. Mayo Clin Proc Digit Health (2025) 3:100257. 10.1016/j.mcpdig.2025.100257 41050187 PMC12492233

[16] Bidgood WD Jr Horii SC Prior FW Van Syckle DE . Understanding and using DICOM, the data interchange standard for biomedical imaging. J Am Med Inform Assoc (1997) 4:199–212. 10.1136/jamia.1997.0040199 9147339 PMC61235

[17] Abbasi AB Layden J Gordon W Gregurick S DeLew N Grossman J A unified approach to health data exchange: a report from the US DHHS. JAMA (2025) 333:1074–9. 10.1001/jama.2025.0068 39826091 PMC11936714

[18] Mandl KD Gottlieb D Mandel JC Ignatov V Sayeed R Grieve G Push button population health: the SMART/HL7 FHIR bulk data access application programming interface. NPJ Digital Medicine (2020) 3:151. 10.1038/s41746-020-00358-4 33299056 PMC7678833

[19] HL7® FHIR. (2025). Available online at: https://www.healthit.gov/topic/standards-technology/standards/fhir.

